# Complete Genome Sequence of Lagilactobacillus salivarius VHProbi A17, Isolated from Fecal Samples

**DOI:** 10.1128/mra.00862-22

**Published:** 2022-11-03

**Authors:** Hongchang Cui, Xinping Bu, Zhi Duan

**Affiliations:** a Qingdao Vland Biotech Co., Ltd., Qingdao, China; Wellesley College

## Abstract

The Lagilactobacillus salivarius strain VHProbi A17 is a bacterium that is found to reside in the intestinal tract. Whole-genome-sequencing-based analysis is an ideal approach for evaluating its probiotic properties. Here, we report the whole-genome sequence of this strain, which contains a chromosome and four plasmids, with GC contents of 32.86%.

## ANNOUNCEMENT

One gram of infant feces was serially diluted in saline solution, and a 10^−8^ dilution was spread onto a de Man-Rogosa-Sharpe (MRS) agar plate. All procedures were in accordance with the Declaration of Helsinki. The inoculum was anaerobically incubated at 37°C for 48 h. A colony designated VHProbi A17 was picked and identified as the species Lagilactobacillus salivarius using 16S rRNA gene sequencing, as described by Fu et al. (1). The strain was sequenced with Illumina HiSeq 2500 and Pacific Biosciences (PacBio) RS II platforms. A single colony was inoculated into 200 mL of MRS broth and cultured at 37°C for approximately 12 h at 150 rpm. The cell biomass was harvested after 10 min of centrifugation at 12,000 × *g*. Total DNA was extracted using the Wizard genomic DNA purification kit (Promega) for both Illumina and PacBio library preparation. For Illumina sequencing, DNA samples were sheared into 400-bp fragments and prepared for library building using the NEXTflex rapid DNA-sequencing kit. The library was used for paired-end Illumina sequencing (2 × 150 bp). For PacBio sequencing, DNA was centrifuged in g-TUBES (Covaris, MA) to obtain fragments. Then, the fragments were purified, end repaired, and ligated with SMRTbell adapters. The resulting sequencing libraries were purified three times using 0.45× volumes of Agencourt AMPure XP beads (Beckman Coulter Genomics, MA). A ~10-kb-insert library was prepared for PacBio sequencing. Finally, 7,127,900 raw reads and 7,041,008 clean reads were generated to reach a depth of 530.72-fold coverage using the Illumina platform. There were 298,634 raw reads (*N*_50_, 233,237 bp) generated using the PacBio platform. The Illumina raw reads were quality filtered with Sickle v1.33 (https://github.com/najoshi/sickle). The PacBio raw reads were processed using SMRT Analysis v2.3.0 to discard adapters and low-quality reads. Then, the clean short and long reads were coassembled to construct complete genomes using Unicycler v0.4.8 (2). Error correction of the PacBio assembly results was performed with Pilon v1.22 (3) using the Illumina reads. If there was a 5,000-bp overlap at the two ends, then the sequence was circularized and one end of the overlap was cut off. The final assembly generated a seamless chromosome and four plasmids. Glimmer v3.02 (4), tRNA-scan-SE v2.0 (5), and barrnap v0.9 (https://github.com/tseemann/barrnap) were used to predict the protein-coding genes, tRNA genes, and rRNA genes, respectively. Genome annotations were performed using the NCBI Prokaryotic Genome Annotation Pipeline (PGAP) v6.2 (6). Default parameters were used for all software unless otherwise specified.

The complete genome sequence of L. salivarius VHProbi A17 contains a 1,746,523-bp circular chromosome and four circular plasmids with GC contents of 32.86%. There were 1,837 protein-coding genes, 22 rRNA genes, 78 tRNA genes, 4 noncoding RNA genes, and 22 pseudogenes in the genome. The characteristics of the plasmids are presented in Table 1.

**TABLE 1:** Summary of data for the plasmids

Plasmid name	Size (bp)	Plasmid type	No. of genes	GenBank accession no.
pA17A	247,948	Circular	240	CP097166
pA17B	27,293	Circular	28	CP097167
pA17C	3,349	Circular	3	CP097168
pA17D	2,898	Circular	2	CP097169

### Data availability.

The whole-genome sequence of L. salivarius VHProbi A17 has been deposited in GenBank with accession numbers CP097165, CP097166, CP097167, CP097168, and CP097169, SRA accession numbers SRR21021025 and SRR21021024, BioProject accession number PRJNA835515, and BioSample accession number SAMN28104644.
